# Complete Genome Sequences of Nine Mycobacteriophages

**DOI:** 10.1128/genomeA.00181-14

**Published:** 2014-05-29

**Authors:** Jorgelina Judith Franceschelli, Cristian Alejandro Suarez, Lucrecia Terán, Raúl Ricardo Raya, Héctor Ricardo Morbidoni

**Affiliations:** a Laboratorio de Microbiología Molecular, Cátedra de Microbiología, Facultad de Ciencias Médicas, Universidad Nacional de Rosario, Rosario, Argentina; b Centro de Referencia para Lactobacilos (CERELA-CONICET), San Miguel de Tucumán, Argentina

## Abstract

Genome analyses of a large number of mycobacteriophages, bacterial viruses that infect members of the genus *Mycobacterium*, yielded novel enzymes and tools for the genetic manipulation of mycobacteria. We report here the complete genome sequences of nine mycobacteriophages, including a new singleton, isolated using *Mycobacterium smegmatis* mc^2^155 as a host strain.

## GENOME ANNOUNCEMENT

Not only have mycobacteriophages been an essential source of materials for the development of genetic tools that allow for mycobacterial genetic manipulation, but also they have proven to be a valuable resource for understanding the molecular evolution of those viruses. Until now, >3,600 mycobacteriophages have been isolated, of which about 600 genomes have been sequenced (see http://www.phagesdb.org), annotated, and characterized. The analysis of those sequences has revealed impressive phage diversity, generating 21 clusters (A·U) of which the phages belonging to the same group have at least 50% nucleotide similarity (1). Eight mycobacteriophages are considered singletons because they did not show enough sequence similarity to be part of the clusters described, suggesting that the genome diversity of this group is not exhausted (2, 3).

We isolated >40 novel mycobacteriophages from soil samples of several geographic locations in Argentina using *Mycobacterium smegmatis* mc^2^155 as a host; we previously reported the characterization of 18 of them. Several of these phages infected *Mycobacterium tuberculosis*, opening the possibility for their use for diagnostic purposes; at the same time, we found an uncommon mechanism for phage heritance represented by partition genes similar to those of plasmids (4). We report here the genome sequences of nine mycobacteriophages (20ES, CRB1, 40AC, Jolie1, Hosp, 39HC, 40BC, Jolie2, and 32HC), all of them belonging to the *Siphoviridae* class, with genome sizes ranging from 44 kbp to 71 kbp. Mycobacteriophage genome sequencing was performed at the Instituto de Agrobiotecnología de Rosario (INDEAR), Argentina, by whole-genome shotgun sequencing using a Life Sciences GS-FLX 454 sequencer. When the genomes had defined ends, these were determined by PCR and Sanger sequencing of the product, as previously reported (4). Phage genome annotation of open reading frames was performed using the DNA Master program, which includes GeneMark version 2.0, Glimmer version 3.02, and Aragorn version 1.1 (available at http://cobamide2.bio.pitt.edu), while probable function assignment was done with HHpred and Pfam (5–8). Three mycobacteriophages, 20ES, CRB1, and 40AC, belong to cluster A according to Hatfull’s classification, while the four members of cluster B, Jolie1, Hosp, 39HC, and 40BC, are highly similar to mycobacteriophage KayaCho (2), except for a few open reading frames (ORFs) not showing any homology to KayaCho. Jolie2 is a member of cluster G, displaying a slightly different G+C content (68% versus 66%), which may reflect the fact that this cluster contains few members, and therefore, subclustering has not yet been proposed. Thus, Jolie2 may represent a different subcluster. As expected, the majority of new isolates belong to the most populous clusters described to date (A and B). Interestingly, mycobacteriophage 32HC is a new singleton, being a temperate phage with an open reading frame (*orf47*) encoding a putative integrase belonging to the tyrosine recombinase family. Bioinformatics analysis suggested a possible tRNA gene (MSMEG_5758) for the integration of this mycobacteriophage into the *M. smegmatis* chromosome. Roughly 50% of the *orf* genes of 32HC displayed homology to *Mycobacterium abscessus* genes encoding hypothetical proteins of unknown function; however, preliminary experiments indicated that 32HC does not infect this mycobacterial species.

### Nucleotide sequence accession numbers.

The complete genomes of these nine mycobacteriophages have been deposited in GenBank under the following accession no.: KJ028219 (32HC), KJ192196 (40AC), KJ410132 (20ES), KJ410133 (Jolie2), KJ410134 (CRB1), KJ433974 (Hosp), KJ433973 (39HC), KJ433975 (40BC), and KJ433976 (Jolie1).
